# Changes of Serum IgG Glycosylation Patterns in Primary Biliary Cholangitis Patients

**DOI:** 10.3389/fimmu.2021.669137

**Published:** 2021-06-25

**Authors:** Xiaoli Zeng, Siting Li, Shiyi Tang, Xi Li, Guoyuan Zhang, Mengtao Li, Xiaofeng Zeng, Chaojun Hu

**Affiliations:** ^1^ Department of Rheumatology, Peking Union Medical College Hospital, Peking Union Medical College & Chinese Academy of Medical Sciences, National Clinical Research Center for Dermatologic and Immunologic Diseases (NCRC-DID) Key Laboratory of Rheumatology & Clinical Immunology, Ministry of Education, Beijing, China; ^2^ Affiliated Hospital of North Sichuan Medical College, Nanchong, China; ^3^ Department of Clinical Laboratory, First Affiliated Hospital of Guangxi Medical University, Nanning, China; ^4^ Department of Laboratory Medicine, North Sichuan Medical College, Nanchong, China

**Keywords:** primary biliary cholangitis, immunoglobulin G, lectin microarray, glycosylation, autoantibody

## Abstract

**Objective:**

Primary biliary cholangitis (PBC) is an autoimmune cholestatic liver disease whose diagnosis is based significantly on autoantibody detection. This study aims to investigate the glycosylation profile of serum IgG in PBC patients using high-throughput lectin microarrays technology.

**Method:**

Lectin microarray containing 56 lectins was used to detect and analyze the expression of serum IgG glycosylation in 99 PBC patients, 70 disease controls (DCs), and 38 healthy controls (HCs). Significant differences in PBC from control groups as well as across PBC subgroups positive for various autoantibodies were explored and verified by lectin blot technique.

**Results:**

Lectin microarray detection revealed that compared to DC and HC groups, the specific glycan level of serum IgG sialic acid in PBC patients was increased. For each PBC subgroup, glycan levels of IgG mannose and galactose were decreased in AMA-M2 positive PBC patients compared to the AMA-M2 negative group. IgG N-Acetylgalactosamine (GalNAc) and fucose were decreased in anti-sp100 positive patients. IgG galactose was increased in anti-gp210 positive patients. IgG mannose was decreased in ACA-positive patients. Although the difference in overall sialic acid level was not observed using lectin blot, all results among the above PBC subgroups were consistent with the results of the technique.

**Conclusion:**

Lectin microarray is an effective and reliable technique for analyzing glycan structure. PBC patients positive for different autoantibody exhibits distinct glycan profile. Altered levels of glycosylation may be related to the occurrence and development of the disease, which could provide a direction for new biomarker identification.

## Introduction

Primary biliary cholangitis (PBC) is a chronic autoimmune cholestatic liver disease characterized by progressive non-suppurative inflammation and destruction of the small and medium-sized bile ducts, resulting in fibrosis, cirrhosis, and eventual liver failure (1, 2). With increasing understanding of the disease, the application of convenient autoantibody detection kits, and changing environmental factors, the incidence and prevalence of PBC have been increasing (3, 4). Previous researches had shown that Chinese patients with PBC had significant morbidity and mortality (5, 6).

The diagnosis of PBC depends largely on the detection of serum autoantibodies in patients. So far, more than sixty different autoantibodies have been found in patients with PBC (7). The serological hallmark of primary biliary cirrhosis is the antimitochondrial antibody (AMA), a highly disease-specific antibody identified in about 90–95% of patients with primary biliary cirrhosis. AMA-positivity constitutes one of the three major diagnostic criteria for PBC (8). There are nine subtypes of AMA, of which anti-M2 is the most important subtype used in routine diagnostic tests for PBC. Although the sensitivity and specificity of AMA detection for PBC are both >90%, it has been reported that 5–10% of patients with PBC were not positive for AMA (9). In addition, AMA can be detected in other autoimmune diseases such as primary Sjögren’s syndrome (PSS), autoimmune hepatitis (AIH) (10, 11), and some infectious diseases like tuberculosis (12). Antinuclear antibodies (ANAs) are present in approximately 50% of PBC patients, and their prevalence in AMA-negative sera reached approximately 85% (13). In the ANA category, research has shown that antinuclear dot antibodies sp100 and antinuclear pore antibodies gp210 are specific biomarkers in PBC serum (9, 14, 15), and they can reduce AMA-negative cases by less than 5% in PBC (16). The sensitivity of anti-sp100 and anti-gp210 for PBC was reported to be approximately 25% (9, 15). Findings indicate that anti-SP100 antibody has an important diagnostic role in AMA-negative PBC patients, while anti-gp210 has the best predictive value regarding progression to end-stage hepatic failure (7, 13). Anti-centromere antibodies (ACA) are important diagnostic markers of systemic sclerosis (SSc) and have been found in other autoimmune diseases. In PBC patients, the ACA positivity rate is about 30%. Several studies indicate that ACA positivity in patients with PBC has significant predictive value for progression to portal hypertension (17, 18). Although the detection of PBC-specific ANA may help to diagnose PBC (19), these ANA, like AMA, have been found in other autoimmune diseases. Therefore, new approaches to identifying more specific biomarkers in PBC are needed (20).

Glycosylation is one of the most common post-translational modifications during protein biosynthesis of proteins, which can have a profound impact on the structure and function of proteins (21). Numerous studies have confirmed that glycans have been recognized as biomarkers for cancer and autoimmune diseases (22–24). Immunoglobulin G (IgG) is the most abundant glycoprotein in human serum. In recent years, a large number of studies have confirmed that IgG glycosylation abnormalities play an important role in the occurrence and development of autoimmune diseases (23, 25, 26). For instance, the lack of terminal sialic acid and galactose residues in IgG glycans are related to anti-inflammatory activity and may precede the onset of disease. Thus, they can be used as a biomarker for the early diagnosis of a disease or a potential target for its treatment (27, 28). In this study, we first used an emerging technology—lectin microarray, which allows high-throughput, high-speed, and highly specific research on aberrant glycosylation—to analyze the expression profile of serum IgG glycosylation in patients with PBC, autoimmune disease controls, and healthy controls, then combined with lectin blot technology for verification, to obtain PBC serum IgG glycosylation expression spectrum.

## Materials and Methods

### Serum Samples

All serum samples involved in the study were collected at Peking Union Medical College Hospital during the period from 2006 to 2019. A total of 207 serum samples collected from 99 PBC patients; 30 AIH patients, 40 PSS patients, and 38 healthy controls (HCs) were used for lectin microarray analysis. Patients with PBC were divided into five subgroups according to their positivity for different autoantibodies. AIH patients and PSS patients constituted the disease controls (DCs). Sample characteristics were presented in **Table 1**. In the lectin blot verification analysis, a new cohort of samples was collected from 55 PBC patients, 16 DC patients (four AIH, twelve PSS), and 16 healthy controls. All patients with PBC were diagnosed according to the criteria established by the American Association for the Study of Liver Diseases (29), while all patients with AIH and PSS were diagnosed according to respective general criteria used for each disease (30, 31). Patients with other autoimmune diseases, cancers, infections, or any severe comorbidities were excluded. In addition, AMA-M2 was detected using the anti-M2–3E ELISA kit (Euroimmune); sp100 and gp210 were detected by line immunoassay (YHLO Biotech Co.), while ACA was detected by indirect immunofluorescence assay with Hep2 cells (Euroimmune). Serum samples were obtained by separation from peripheral blood and stored at −80°C until use. The study was approved by the ethics committee at PUMCH and fulfilled the ethical guidelines of the declaration of Helsinki.

**Table 1:** Sample characteristics and subgroups of lectin microarray cohort.

	PBC (n = 99)					DC (n = 70)		HC (n = 38)
	AMA-M2+ Group A	AMA-M2– Group B	sp100+ Group C	gp210+ Group D	ACA+ Group E	AIH	PSS	
n	20	20	19	20	20	30	40	38
Male/Female	1/9	3/17	1/18	1/19	1/19	1/14	1/9	3/35
Age (Mean ± SD)(year)	52.40 ± 10.62	53.35 ± 11.14	56.89 ± 10.72	50.70 ± 7.97	54.55 ± 12.04	44.86 ± 19.28	48.52 ± 9.73	45.60 ± 7.64
AMA-M2 autoantibody	+	–	+	+	+	NA	NA	NA
Anti-sp100 autoantibody	–	–	+	–	–	NA	NA	NA
anti-gp210 autoantibody	–	–	–	+	–	NA	NA	NA
ACA autoantibody	–	–	–	–	+	NA	NA	NA

PBC, primary biliary cholangitis; DC, disease control; AIH, autoimmune hepatitis; PSS, primary Sjögren’s syndrome; HC, healthy control; AMA-M2, antimitochondrial antibody M2; gp210, anti-glycoprotein-210 antibody; sp100, sp100 nuclear antigen; ACA, anti-centromere antibody.

### Sera Assay With Lectin Microarray

A commercial lectin microarray (BCBIO Biotech, Guangzhou, China) with 56 lectins was used to detect the glycopattern of serum IgG, which can quickly and sensitively detect common glycan variants in IgG. First, lectin microarrays were removed from −80°C and warmed at room temperature for half an hour, then incubated with blocking buffer (3% BSA in PBS) at room temperature for 2 h. Microarrays were then washed with PBS and dried by spinning at 500 g for 5 min. Subsequently, 200 µl of the 1:1,000 diluted samples sera were applied to the microarray and incubated overnight at 4°C. After washing three times with PBS, the microarrays were incubated with 5 ml Cy5-labeled goat anti-mouse IgG antibody (1:1,000; Jackson Laboratory, Bar Harbor, ME) in the dark at room temperature for 50 min. Finally, after three washes with PBS and two washes with D.I. water, the microarrays were dried by spinning at 500 g for 5 min and scanned using the GenePix 4000B (Molecular Devices, Sunnyvale, CA) Microarray Scanner at a wavelength of 635 nm and a photomultiplier tube setting of 600.

### Lectin Microarray Data Analysis

For lectin array assays, the GenePix Pro 6.0 software (Molecular Devices, Sunnyvale, CA) and proprietary gal files were used to extract the median foreground and background intensity values for each spot on the arrays. The signal-to-noise ratio (S/N) (the medium intensity of the spot foreground relative to the background) of each lectin spot was calculated. To reduce the bias of the lectin microarray in the inter-array, we normalized the S/N data in terms of Between Arrays (32). In addition, we determined that there were significant differences in lectin binding between the test groups by using the method of Hu et al. (33), and for the difference, lectin must meet the following two conditions: (a) fold change [group1 (S/N)/group2 (S/N)] ≥1.3 or <0.77, (b) *P* value <0.05.

### Lectin Blot Analysis

To validate the results of the lectin microarray, 60 PBC patients, 12 DCs (five AIH, seven PSS), and 12 HCs randomly chosen from the lectin microarray analysis cohort, as well as 55 PBC patients, 16 DCs (four AIH, twelve PSS), and 16 HCs from a new cohort were selected for lectin blotting analysis. Briefly, to determine the location of IgG in immunoblotting, 1:100 diluted serum proteins were separated by 10% sodium dodecyl sulphate–polyacrylamide gel electrophoresis (SDS–PAGE). The separated proteins were electrotransferred onto polyvinylidene fluoride membranes (Millipore, Billerica, MA). After blocking non-specific binding sites with 10× Carbo-Free Blocking Solution (1:10; Vector Laboratories Inc., US) at room temperature for 2 h, the membranes were incubated with horseradish peroxidase-conjugated anti-human IgG (1:2,000) at room temperature for 1 h. The reactive bands were visualized by ImageQuant (GE Healthcare). Then, lectin blot was used to verify the binding of serum to lectins. As was mentioned above, after blocking non-specific binding sites, the membranes were incubated with Cy3 (1:1,000; GE Healthcare)-labeled lectins including SSA, ABA, LAL, ConA (EY Laboratories, Inc., US), GNL, SBA, BPL, PSA (Vector Laboratories Inc. US) at 4°C overnight in the dark. Excess lectins were removed by washing three times with PBST. The washed and dried membranes were detected by a fluorescence signal system of Typhoon FLA 9500 (GE Healthcare). Finally, ImageJ software was used for signal intensity analysis.

### Statistical Analysis

**S**PSS 22.0 was used to perform all statistical analyses, and GraphPad Prism 8 was used to drew plots in the study. Continuous variables were expressed as mean ± standard deviation. The differences among the PBC, DC, and HC groups were tested by one-way analysis of variance (ANOVA); the Student t-test was used to compare the antibody-positive group with the negative group in PBC patients. *P* value less than 0.05 was considered statistically significant.

## Results

### Analysis of Serum IgG Glycosylation in Patients With PBC by Lectin Microarray

**Figure 1:**
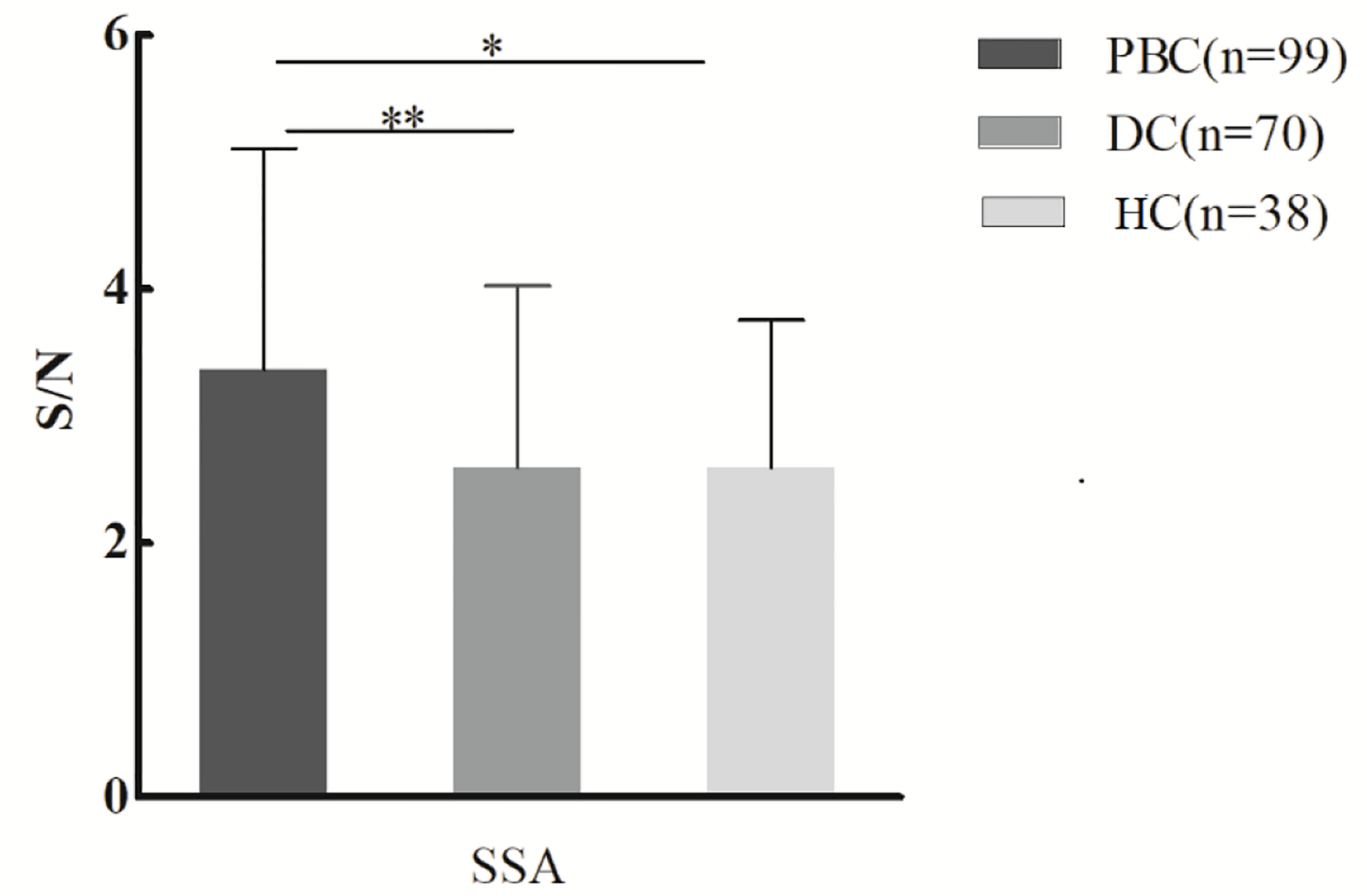
Specific changes of serum IgG glycosylation in PBC patients’ comparison with DCs and HCs from the lectin microarray. *P < 0.05. PBC, primary biliary cholangitis; DC, disease control; HC, healthy controls; SSA, *Salvia sclarea*. S/N, the medium intensity of the spot foreground relative to the background. **p < 0.01.

Serum samples of 99 PBC patients, 70 DC patients, and 38 healthy controls were detected by lectin microarray. As shown in **Table 2**, after signal normalization, there were five lectins among 56 lectins that showed differential signal intensities between the PBC and HC groups (*P*< 0.05), while one was significantly different between the PBC and DC groups (*P* < 0.05). Compared with the DC and HC groups, serum IgG from PBC patients had a higher affinity for SSA. Therefore, glycan level of sialylation (recognized by SSA) was increased characteristically in serum IgG from patients with PBC (**Figure 1** **)**. Thus, SSA was chosen for verification in the later process.

**Table 2:** Difference of specific binding between IgG and lectin in PBC, DC, and HC groups.

Lectin	Normalized fluorescence intensity (Mean ± SD)			Fold change	
	PBC	DC	HC	PBC/DC	PBC/HC
SSA	3.37 ± 1.74	2.60 ± 1.43	2.60 ± 1.43	1.30^**^	1.30^*^
ABA	2.27 ± 1.16	1.95 ± 0.86	1.63 ± 0.49	1.16	1.39^**^
RCA-I	3.90 ± 2.01	3.31 ± 1.73	2.89 ± 1.13	1.18^*^	1.35^**^
ACL	1.60 ± 0.91	1.85 ± 1.19	2.39 ± 3.06	0.87	0.67^*^
SNA-I	6.13 ± 4.50	5.61 ± 3.68	4.45 ± 2.54	1.09	1.38^*^

**P < 0.01, *P < 0.05; PBC, primary biliary cholangitis; DC, disease control; HC, healthy control; SSA, Salvia sclarea; ABA, Agaricus bisporus lectin; RCA-I, Ricinus communis agglutinin I; ACL, Amaranthus caudatus lectin; SNA-I, Sambucus nigra.

**Figure 2:**
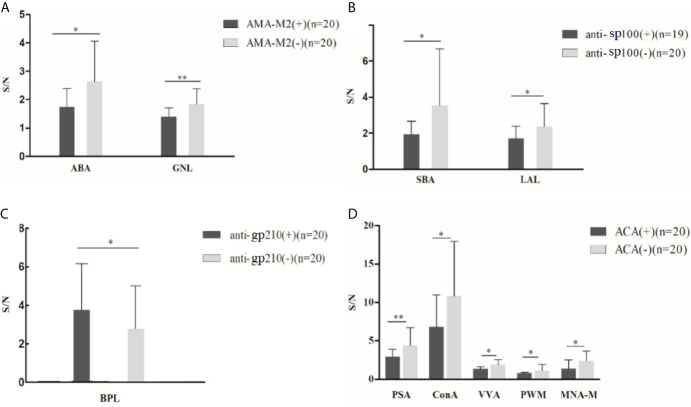
Binding levels of specific lectins between autoantibody-positive and negative groups in PBC patients from lectin microarray. **(A)** Comparison of serum IgG glycosylation between AMA-M2 positive and negative groups (group A and group B); **(B)** Comparison of serum IgG glycosylation between sp100 positive and negative groups (group C and group A); **(C)** Comparison of serum IgG glycosylation between gp210 positive and negative groups (group D and group A); **(D)** Comparison of serum IgG glycosylation between ACA positive and negative groups (group E and group A). *P < 0.05,**P < 0.01. ABA, *Agaricus bisporus* lectin; GNL, *Galanthus nivalis* lectin; SBA, glycine max lectin; LAL, *Laburnum anagyroides* lectin; BPL, *Bauhinia purpurea* lectin; PSA, *Pisum sativum* agglutinin; ConA, concanavalin A lectin; VVA, *Vicia villosal* lectin; PWM, *Phytolacca americana* lectin; MNA-M, *Morniga M* lectin. S/N, the medium intensity of the spot foreground relative to the background.

Lectin microarray results were further explored across different PBC subgroups (**Table 1**), and results were illustrated in **Figure 2**: (1) Significantly lower glycan levels of galactose (recognized by ABA) and mannose (recognized by GNL) were observed for AMA-M2 positive patients compared to the negative group (*P* < 0.05). (2) Significantly lower glycan levels of N-Acetylgalactosamine (GalNAc) (recognized by SBA) and fucose (recognized by LAL) were observed for anti-sp100 positive patients compared to the negative group (*P* < 0.05). (3) Significantly higher glycan level of galactose (recognized by BPL) was observed for anti-gp210 positive patients compared to the negative group (*P* < 0.05). (4) Significantly lower glycan levels of mannose (recognized by PSA, Con A, VVA, and MNA-M) and GlcNAc (recognized by PWM) were observed for ACA-positive patients compared to the negative group (*P* < 0.05).

### Validation of Glycosylation Changes of IgG by Lectin Blot

**Figure 3:**
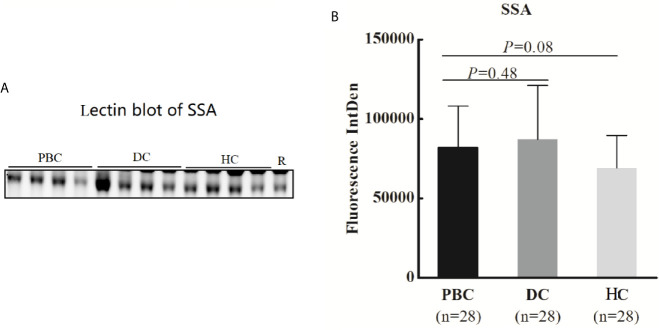
Lectin blot of SSA lectin for serum IgG in PBC, DC and, HC groups. **(A)** Lectin blot of SSA for serum IgG from three groups, 12 PBC patients, 12 DCs (five AIH, seven PSS), and 12 HCs was selected from lectin microarray cohort, 16 PBC patients,16 DCs (four AIH, twelve PSS), and 16 HCs were selected from a new cohort. **(B)** Comparison of fluorescence intensity of lectin blot bands in PBC, DC, and HC groups. PBC, primary biliary cholangitis; DC, disease control; HC, healthy controls; SSA, *Salvia sclarea*; R, reference.

IgG heavy chains were selected in lectin blot to verify the microarray results. The intensity of SSA on serum IgG from 28 PBC patients, 28 DC patients, and 28 healthy controls was analyzed, and no significant results were observed (**Figure 3**).

**Figure 4:**
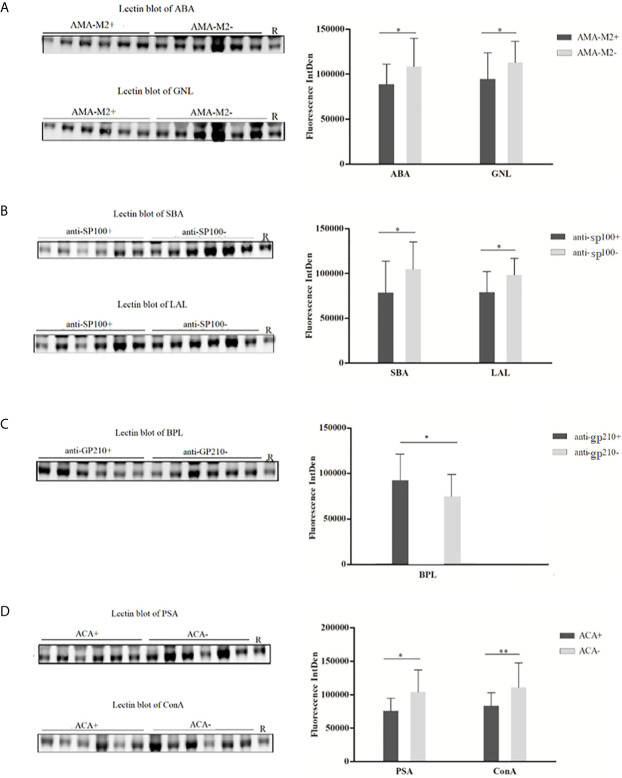
Lectin blot of selected lectins for serum IgG between PBC subgroups. **(A)** Lectin blots for ABA and GNL and band fluorescence intensity between AMA-M2 positive and negative groups. **(B)** Lectin blots for SBA and LAL, and band fluorescence intensity between anti-sp100 positive and negative groups. **(C)** Lectin blots for BPL and band fluorescence intensity between anti-gp210 positive and negative groups. **(D)** Lectin blots for PSA and ConA and band fluorescence intensity between ACA positive and negative groups. *P < 0.05, **P < 0.05. ABA, *Agaricus bisporus* lectin; GNL, *Galanthus nivalis* lectin; SBA, glycine max lectin; LAL, *Laburnum anagyroides* lectin; BPL, *Bauhinia purpurea* lectin; PSA, *Pisum sativum* agglutinin; ConA, concanavalin A lectin; R, reference.

For each PBC subgroup, 16 serum samples (six from lectin microarray cohort and ten from a new cohort, randomly selected from subgroups positive for respective autoantibodies) were chosen for validation. The results were listed as follows (**Figure 4**): (1) ABA and GNL lectins were selected to recognize glycans of serum IgG in AMA-M2 negative and positive groups, and decreased levels of galactose and mannose in AMA-M2 positive group were observed (*P* < 0.05). (2) SBA and LAL lectins were selected to recognize glycans of serum IgG in anti-sp100 negative and positive groups, and decreased levels of GalNAc and fucose in anti-sp100 positive group were observed (*P* < 0.05). (3) BPL was selected to recognize glycans of serum IgG in anti-gp210 negative and positive groups, and increase of galactose in anti-gp210 positive group was observed (*P* < 0.05). (4) PSA and ConA lectins were selected to recognize glycans of serum IgG in ACA negative and positive groups, and decrease of mannose in ACA positive group was observed (*P* < 0.05). These results were consistent with those from lectin microarrays, which confirmed the reliability of lectin microarray analysis. The consistent summary of verification results was shown in **Table 3**.

**Table 3:** Significant microarray result for changes of serum IgG glycan in PBC subgroups.

Subgroup	Lectin	Binding polysaccharide	Positive group result
AMA-M2	ABA	galactose	decreased
	GNL	mannose	
Anti-sp100	SBA	GalNAc	decreased
	LAL	fucose	
Anti-gp210	BPL	galactose	increased
ACA	PSA	mannose	decreased
	Con A	mannose	

AMA-M2, antimitochondrial antibody M2; gp210, anti-glycoprotein-210 antibody; sp100, sp100 nuclear antigen; ACA, anti-centromere antibody; ABA, Agaricus bisporus lectin; GNL, Galanthus nivalis lectin; SBA, glycine max lectin; LAL, Laburnum anagyroides lectin; BPL, Bauhinia purpurea lectin; PSA, Pisum sativum agglutinin; ConA, concanavalin A lectin; GalNAc, N-acetylgalactosamine.

## Discussion

IgG is the most abundant antibody in our body and was involved in multiple humoral immune processes: antigen neutralization, complement activation, antibody-dependent cell-mediated cytotoxicity (ADCC), and complement-dependent cytotoxicity (CDC), *etc.* (28, 34). The Fc fragment of IgG plays an important role in some of these processes, and variation of Fc glycans composition could influence IgG’s structural, conformation, and effector functions (35, 36). To date, a large number of studies have reported that IgG glycosylation patterns and expression levels have changed in many different autoimmune diseases such as rheumatoid arthritis (RA), systemic lupus erythematosus (SLE), granulomatous vasculitis (GPA), and inflammatory bowel disease (IBD) (37–40). Considering the important role of IgG glycosylation in autoimmune diseases, this study explored the patterns and levels of IgG-specific glycosylation in PBC patients and subgroups positive for different autoantibodies.

Lectin microarray is an emerging technique for the study of glycosylation in recent years (41, 42). Compared with other conventional polysaccharide analysis methods such as mass spectrometry, it enables rapid, high-throughput, and high-sensitivity profiling of complex glycan features to directly obtain global glycosylation without the need for liberation of glycans (43, 44). Lectin microarray has made important achievements in the study of glycosylation and biomarker identification of tumors and autoimmune diseases (22, 45, 46). In our study, a total of 207 serum samples were analyzed by lectin microarray. The fluorescence intensity of specific binding of serum IgG to 56 lectins had increased and decreased alteration, which indicated that these results were related to the structure of serum IgG polysaccharides, but not to the increase of serum IgG concentration. Compared with the DC and HC groups, serum IgG from PBC patients had a higher affinity for SSA lectin detected by lectin microarray, which was not consistent with the result of lectin blotting of SSA. It might be due to the limited number of tested specimens or excessive heterogeneity. However, for PBC subgroups positive for different antibodies, the lectin microarray results were all consistent with corresponding lectin blot verification results. Our study suggested that lectin microarray could be an alternative method in identifying PBC patients apart from diagnostic autoantibodies and have additive value for autoantibody-negative patients.

Serum AMA-M2 is considered a pivotal biomarker for the diagnosis of PBC. In our study, the results had shown that galactose and mannose were lower in AMA-M2 positive patients than those observed for AMA-M2-negative patients. A decrease in the level of IgG galactosylation has been found in various autoimmune diseases, such as RA, SLE, autoimmune vasculitis, and Crohn’s disease. In a previous study using mass spectroscopy, Zhou et al. observed a decrease of terminal galactosylation in PBC and PSC patients (47). Early studies have proposed that IgG which lacks terminal galactoses might have an increased level of pro-inflammatory activity through MBL-dependent pathway of complement activation (26, 28). As the role of AMA-M2 in PBC is still controversial, these changes of serum IgG glycosylation may be helpful in analyzingits role in PBC disease.

Anti-nuclear antibodies to speckled 100 kDa (sp100) are highly specific to PBC and can be detected in 10–40% of PBC patients (19, 48). Compared to sp100-negative patients, the expressions of GalNAc and fucose were decreased in anti-sp100 positive PBC patients in our study. Previous studies have shown that the majority (>90%) of serum IgG contains a fucose which was attached to the first GlcNAc in the IgG glycan core structure (49, 50). Reduced core fucosylation of the IgG promotes increased activating Fc*γ*R signaling and enhances inflammatory effector cell activity (51, 52). The presence of a high proportion of fucose for IgG can reduce potentially harmful ADCC activity (26). Therefore, we speculate that changes of serum IgG glycosylation in patients with positive anti-sp100 antibodies may be involved in the occurrence of PBC disease.

Anti-gp210 antibodies, one of the PBC-specific antinuclear antibodies (ANAs), are highly specific for PBC and can be detected in 20–30% of PBC patients (53). Several studies have indicated that autoantibodies of gp210 are strong predictors of PBC prognosis (54, 55). In addition, faster disease progression, and significantly higher hepatic failure have been found in anti-gp210-positive patients (56, 57). In this study, we have observed that the expression of galactose was increased in anti-gp210-positive patients with PBC. IgG galactosylation can change quickly in acute inflammation. Highly galactosylated immune complexes have been reported to inhibit the pro-inflammatory activity of complement component C5a (58). Likewise, terminal galactose has been found to enhance the affinity of IgG to complement C1q and Fc*γ* receptors, thus promoting CDC and ADCC (59, 60). Therefore, the changes of serum IgG glycosylation in anti-gp210 positive patients may play an important role in the occurrence of PBC or may reflect the inflammatory state of the disease.

Anti-centromere (ACA) antibodies are also a kind of antinuclear antibody which can be detected in 25–30% of PBC patients (7). Previous study has revealed an elevated rate of annual estimated glomerular filtration rate (eGFR) declines in PBC patients with ACAs compared to those without ACAs, which may be associated with the vascular damage due to anti-endothelial cell antibodies (AECAs). Thus, ACAs may be an independent predictor for chronic kidney disease (CKD) in PBC (61, 62). Our study demonstrates that the expression of mannose was decreased in ACA positive PBC patients, which may be related to the pathogenesis and influence of PBC.

Our study has some limitations. Inconsistency between the results of serum IgG and lectin SSA in patients with PBC detected by the two techniques may be due to the limited number of tested specimens or excessive heterogeneity. In the future, clinical features of patients (baseline characteristics, disease duration, biochemical parameters, *etc.*) could be recorded, controlled, and explored for their relationship with specific glycosylate profiles in a larger cohort.

In conclusion, our study is the first to use lectin microarrays to analyze changes in serum IgG glycosylation patterns in PBC patients. The results show that PBC patients positive for different autoantibodies have specific glycosylation patterns, which provides a new direction and method for us to further explore the pathogenesis and look for new biomarkers of PBC.

## Data Availability Statement

The raw data supporting the conclusions of this article will be made available by the authors without undue reservation.

## Ethics Statement

The studies involving human participants were reviewed and approved by the Ethics Committee of Peking Union Medical College Hospital. The patients/participants provided their written informed consent to participate in this study.

## Author Contributions

All the authors were involved in the design of the project. XLZ, ST, and XL contributed to all of the experimental procedures. GZ and ML contributed significantly to analysis and manuscript preparation. XL and ST analyzed the data and wrote the manuscript. XFZ and CH helped perform the analysis with constructive discussions. All authors contributed to the article and approved the submitted version.

## Funding

This study was supported by the National Key Research and Development Program of China (2019YFC0840603, 2017YFC0907601, and 2017YFC0907602), the National Natural Science Foundation of China (81771780), and the CAMS Initiative for Innovative Medicine (2017-I2M-3-001 and 2019-I2M-2-008).

## Conflict of Interest

The authors declare that the research was conducted in the absence of any commercial or financial relationships that could be construed as a potential conflict of interest.
